# Dynamic vitamin D trajectories and their prognostic value in breast cancer: a group-based trajectory modeling study

**DOI:** 10.3389/fnut.2026.1839196

**Published:** 2026-06-04

**Authors:** Lin-bo Yin, Xue-ling Lan, Tao Chen, Jing Zhang, Yang Zheng

**Affiliations:** Department of Clinical Laboratory, Cancer Hospital of Dalian University of Technology, Liaoning Cancer Hospital and Institute, Shenyang, China

**Keywords:** breast cancer, prognosis, risk stratification, trajectory analysis, vitamin D

## Abstract

Breast cancer represents the most frequently diagnosed malignancy and a leading cause of cancer-related mortality among women globally, with substantial prognostic heterogeneity that underscores the urgent need for robust prognostic biomarkers to guide personalized clinical management. Although accumulating evidence has linked vitamin D status to breast cancer progression, most existing studies have relied on static single measurements of serum vitamin D, leading to inconsistent findings, while the prognostic implications of dynamic longitudinal changes in vitamin D levels during treatment and follow-up remain largely underexplored. In this retrospective cohort study, we enrolled 513 female patients with pathologically confirmed primary invasive breast cancer treated between 2020 and 2023, with a median follow-up duration of 38.0 months. Serial serum 25-hydroxyvitamin D measurements were obtained at three predefined time points: baseline, mid-treatment, and post-treatment follow-up. Group-based trajectory modeling (GBTM) was employed to identify distinct latent subgroups with homogeneous vitamin D change patterns. We then evaluated the association between these trajectories and event-free survival (EFS) using Kaplan–Meier analysis, log-rank test, and multivariable Cox regression, and further constructed a trajectory-based risk stratification model and a visual prognostic nomogram, whose performance was validated via concordance index (C-index), calibration curves, and decision curve analysis. We identified 6 distinct vitamin D trajectories, which were further categorized into three risk strata, with 64.9% of patients presenting with severe vitamin D deficiency at baseline. After multivariable adjustment for established clinical factors, dynamic vitamin D trajectories were confirmed as an independent prognostic factor for EFS: patients in the high-risk group (characterized by persistent deficiency or worsening vitamin D status) exhibited significantly worse survival outcomes compared with the low-risk group with consistently sufficient vitamin D levels (all pairwise *p* < 0.001). Landmark analysis further confirmed the robustness of these findings by ruling out potential survivorship bias. The integrated prognostic nomogram, which incorporated age, tumor stage, molecular subtype, and trajectory risk group, achieved a C-index greater than 0.7, with favorable calibration and substantial clinical net benefit, demonstrating superior discriminative ability compared with traditional prognostic models. In conclusion, dynamic serum vitamin D trajectories serve as a strong, independent prognostic indicator for breast cancer patients. Our findings highlight the clinical significance of longitudinal vitamin D monitoring, and the proposed risk stratification tool provides a practical approach to facilitate individualized prognostic assessment and optimized clinical management for breast cancer patients.

## 1 Introduction

Breast cancer remains the most commonly diagnosed malignancy and a leading contributor to cancer-related mortality among women worldwide, posing a severe threat to global female health (1, 2). Despite advancements in screening, surgical techniques, and systemic therapies including chemotherapy, targeted therapy, and immunotherapy, the prognosis of breast cancer patients remains highly heterogeneous, largely due to the complex interplay of tumor biological characteristics and host-related factors (3, 4). Personalized risk stratification and prognostic assessment have thus become central to optimizing clinical management and improving patient outcomes, highlighting the urgent need to identify novel, reliable, and clinically actionable prognostic biomarkers.

Vitamin D, a fat-soluble secosteroid traditionally recognized for its role in calcium homeostasis and bone metabolism, has emerged as a promising candidate in cancer research due to its pleiotropic biological effects (5, 6). Preclinical studies have demonstrated that the active form of vitamin D, 1,25-dihydroxyvitamin D, exerts anti-tumor effects through multiple mechanisms, including inhibition of cell proliferation, induction of apoptosis, suppression of angiogenesis, and modulation of the tumor microenvironment and immune response (7, 8). Epidemiological and clinical studies have subsequently explored the association between vitamin D status and breast cancer risk and prognosis, yet the results remain inconsistent (9–11). Most existing research has relied on static single measurements of serum 25-hydroxyvitamin D [25(OH)D]—the gold standard for assessing vitamin D status—failing to capture the dynamic changes in vitamin D levels that occur during breast cancer diagnosis, treatment, and follow-up.

Tumor treatment modalities such as surgery, chemotherapy, and radiotherapy can alter host metabolism and nutritional status, while nutritional interventions and lifestyle modifications may also influence vitamin D levels over time. This dynamic variability suggests that a single baseline or post-treatment measurement of vitamin D may not fully reflect the biological interplay between vitamin D and breast cancer progression, potentially explaining the conflicting findings in previous static analyses. GBTM, a powerful statistical approach for identifying distinct longitudinal subpopulations with homogeneous change patterns, offers a novel way to characterize dynamic vitamin D trajectories in breast cancer patients (12, 13). By grouping patients based on serial vitamin D measurements, GBTM can uncover clinically meaningful subtypes of vitamin D status changes and their potential prognostic value.

In this retrospective cohort study, we aimed to (1) identify distinct dynamic trajectories of serum vitamin D levels in a cohort of breast cancer patients over the course of treatment and follow-up using GBTM; (2) evaluate the association between these vitamin D trajectories and EFS, a key prognostic endpoint for breast cancer; (3) verify the independence of this prognostic association after stratification by established clinical factors including tumor stage and molecular subtype; and (4) construct a new vitamin D trajectory risk stratification model and develop a visual nomogram for clinical applications. We hypothesized that dynamic vitamin D trajectories would serve as a stronger and independent prognostic factor than static single measurements, and that integrating longitudinal vitamin D monitoring into clinical practice could enhance risk stratification and guide personalized nutritional intervention strategies for breast cancer patients.

## 2 Materials and methods

### 2.1 Study design

This retrospective cohort study extracted data from the electronic medical record (EMR) system, focusing on adult breast cancer patients who were treated at Liaoning Cancer Hospital between January 1, 2020, and December 31, 2023. The study defined three time points: baseline (Time 1, initial vitamin D measurement at the first visit): reflects pre-treatment nutritional status, which is associated with initial tumor biology (14, 15); mid-treatment (Time 2, 3–6 months post-surgery or after radiotherapy and chemotherapy): Captures the peak impact of cancer treatment on host metabolism, representing the critical window for nutritional intervention (16, 17); and post-treatment or long-term follow-up (Time 3, 6–12 months after treatment): represents stable nutritional status after treatment completion, which correlates strongly with early recurrence risk (18). The primary study variables included demographic and clinical characteristics and serial vitamin D levels. This study was approved by the Institutional Review Board of Liaoning Cancer Hospital. Given the retrospective design and the use of de-identified patient data, the requirement for informed consent was waived. All research activities strictly adhered to the ethical principles outlined in the Declaration of Helsinki.

#### 2.1.1 Inclusion criteria

1. Patients with pathologically confirmed primary female invasive breast cancer;
2. Patients who received standard surgery and postoperative adjuvant chemoradiotherapy or systemic therapy at our hospital with a complete medical record;
3. Patients with complete vitamin D measurements at three time points (baseline, mid-treatment, post-treatment follow-up), as well as complete clinicopathological and follow-up data;
4. Patients whose diagnosis date and follow-up period fell within the study’s predefined time range.

#### 2.1.2 Exclusion criteria

1. Patients with a history of other primary malignant tumors;
2. Patients with severe liver or kidney disease, parathyroid dysfunction, autoimmune diseases, or other comorbidities that affect vitamin D metabolism;
3. Patients who had long-term use of glucocorticoids, antiepileptic drugs, therapeutic-dose vitamin D preparations, or other medications known to affect vitamin D levels before enrollment;
4. Patients with missing follow-up data, loss to follow-up, or incomplete core clinicopathological information;
5. Patients who received neoadjuvant therapy before surgery.

The median follow-up duration in the cohort was 38.0 months (range: 6.0–54.0 months). The overall censoring rate was 72.5%, and the loss to follow-up rate was 3.1%, yielding an analytical cohort of 513 patients.

### 2.2 Vitamin D measurement

This study employed LC–MS/MS to quantify serum vitamin D levels. This method is specifically designed for the quantification of total 25-hydroxyvitamin D in human serum and plasma, including the two main metabolites—25-hydroxyvitamin D2 (25(OH)D2) and 25-hydroxyvitamin D3 (25(OH)D3)—thereby ensuring comprehensive and accurate assessment. To ensure consistency and comparability of analyses, we adopted uniform classification criteria for vitamin D status in this study: serum vitamin D concentrations <20 ng/mL were defined as deficiency, 20–30 ng/mL as insufficiency, and >30 ng/mL as sufficiency (19, 20).

### 2.3 Study endpoints

The primary endpoint was EFS, a composite endpoint integrating disease-free survival (DFS) in patients with oligometastatic disease who received definitive local therapy and progression-free survival (PFS) in patients with widespread metastatic disease who received palliative systemic therapy.

Specifically, EFS was defined as the time interval from the respective index date to the first occurrence of any of the following events:

1. Radiographically confirmed disease recurrence or distant metastasis, in patients who had undergone curative-intent surgical resection or stereotactic body radiation therapy (SBRT) for oligometastatic disease;
2. Radiographically confirmed disease progression according to RECIST version 1.1 criteria, in patients receiving palliative systemic therapy;
3. Death from any cause, regardless of prior documentation of disease progression or recurrence.

The index date was defined as the date of completion of definitive local therapy for patients in the curative subgroup, and the date of randomization for patients in the palliative subgroup. Patients who remained alive and had not experienced any of the above events at the time of their last documented follow-up visit were censored at that date.

Patients who died without prior radiological documentation of disease progression or recurrence due to clinical deterioration were considered to have had an event. Patients who discontinued treatment due to adverse events without subsequent disease progression or death were censored at the time of treatment discontinuation.

### 2.4 Statistical analysis

All statistical analyses were performed using R software (Version 4.3.1) and SPSS 26.0. Continuous variables with normal distribution were presented as mean ± standard deviation (SD) and range, while categorical variables were described as n (%).

Group-based trajectory modeling (GBTM) was performed using the traj package in R to identify distinct longitudinal trajectories of serum vitamin D levels across the three predefined time points (T1, T2, T3). A systematic multi-step model selection and validation workflow was implemented as follows:

1. A priori model specification: We modeled the longitudinal changes of serum vitamin D levels as a cubic polynomial function of time, and sequentially fitted models with 2 to 7 potential trajectory subgroups to avoid underfitting or overfitting.
2. Multi-dimensional model selection criteria: The optimal number of trajectory subgroups was determined by integrating quantitative and qualitative metrics: ① Bayesian Information Criterion (BIC) and log Bayes factor (2ΔBIC), with lower BIC values and 2ΔBIC > 10 indicating strong evidence for superior model fit; ② Entropy value, with values >0.8 indicating high classification precision and clear subgroup distinction; ③ Average Posterior Probability of Assignment (APPA) for each subgroup, with APPA >0.9 indicating excellent individual classification reliability; ④ Clinical interpretability and biological plausibility of the identified trajectory patterns, to ensure the final model conformed to the clinical reality of vitamin D dynamic changes during breast cancer treatment and follow-up.
3. Robustness validation of trajectory classification:1000-times bootstrap resampling was performed to verify the stability of trajectory classification, with the average classification agreement rate between bootstrap resampled models and the original model calculated to quantify classification consistency.
4. Sensitivity analyses: Landmark analysis excluding patients with EFS events within 12 months of follow-up, to rule out the impact of survivorship bias.

EFS was the primary prognostic endpoint, and Kaplan–Meier curves were plotted to depict EFS differences among different vitamin D trajectory subgroups, tumor stages, and molecular subtypes. The Log-rank test was used for inter-group comparison of survival curves, with the Consistently Sufficient vitamin D group set as the reference for trajectory subgroup analyses. A Cox proportional hazards regression model was applied to evaluate the prognostic value of the risk-stratified vitamin D trajectory groups and to construct the prognostic prediction model. The predictive performance of the model was assessed using the concordance index (C-index), where a C-index > 0.7 was defined as good predictive ability. A prognostic nomogram was developed to visualize the combined risk prediction model, integrating core clinical and laboratory predictors. Heatmaps and box plots were generated for descriptive and comparative analysis of the interaction between baseline vitamin D levels and risk-stratified trajectory groups, as well as the distribution of baseline vitamin D levels across different risk subgroups. A two-sided *p* < 0.05 was considered statistically significant for all analyses.

## 3 Results

### 3.1 Patient population

**Figure 1:**
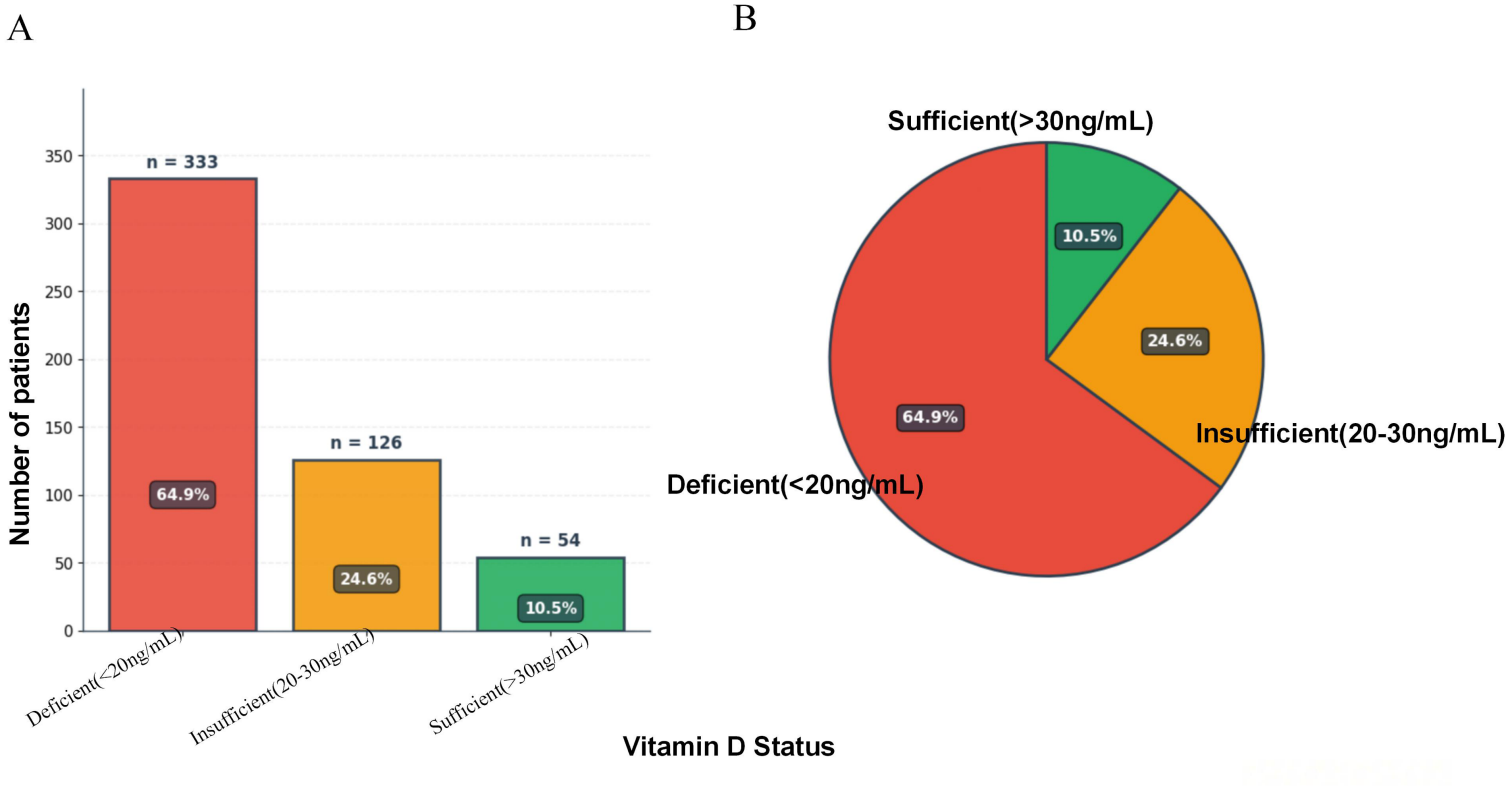
Distribution of vitamin D levels in breast cancer patients at Time 1 (T1). Bar graph **(A)** and pie chart **(B)** presenting the distribution of vitamin D levels in peripheral blood serum at the first detection in breast cancer patients at initial diagnosis.

This study included a total of 513 female breast cancer patients who met the inclusion criteria. The mean age of the patients was 55.8 ± 10.9 years. Additionally, the mean body mass index (BMI) was 25.0 ± 4.6 kg/m^2^. Table 1 summarizes the demographic, clinicopathological characteristics, and vitamin D levels at the three predefined time points (Time 1, Time 2, Time 3). We also compared the baseline characteristics of the included patients (*n* = 513) with the excluded patients due to incomplete vitamin D measurement (*n* = 127), and no statistically significant differences were observed (all *p* > 0.05, Supplementary Table 1). As shown in Figure 1, 64.9% of breast cancer patients (*n* = 333) presented with severe vitamin D deficiency at baseline, a finding that warrants close clinical attention. 89.5% of patients (including those with deficiency and insufficiency, *n* = 459) required vitamin D supplementation, a proportion significantly higher than that of the vitamin D-sufficient group (10.5%, *n* = 54).

**Table 1:** Demographic and clinical characteristics of the clinic cohort.

Variable	Clinic cohort (*n* = 513)
Gender, *n* (%)	
Female	513 (100%)
Age, mean (SD) [range] years	55.8 ± 10.9 [20–91]
Body mass index, mean (SD) [range] kg/m2	25.0 ± 4.6 [16.1–35.8]
Tumor Stage, *n* (%)	
Stage I	122 (23.8%)
Stage II	153 (29.8%)
Stage III	147 (28.7%)
Stage IV	91 (17.7%)
Molecular subtype, *n* (%)	
Luminal A	198 (38.6%)
Luminal B	131 (25.5%)
HER2-positive	95 (18.5%)
Triple-negative breast cancer, TNBC	89 (17.3%)
Vitamin D Level, mean (SD) [range] ng/mL	
Time 1	17.97 ± 8.99 [2.28–64.97]
Time 2	18.48 ± 9.53 [2.57–64.86]
Time 3	18.86 ± 9.66 [2.05–58.23]

### 3.2 GBTM of dynamic vitamin D level changes

Given the prevalent vitamin D deficiency at baseline in the study population, to further explore the dynamic variation patterns of vitamin D levels throughout the entire treatment course and identify subpopulations with characteristic changes, GBTM was applied to analyze vitamin D data at three time points for subtyping, aiming to reveal the longitudinal change characteristics of vitamin D levels.

We sequentially fitted GBTMs specifying 2 to 7 distinct latent trajectory subgroups, with detailed fit metrics for all evaluated models summarized in Supplementary Table 2. Model selection was guided by a pre-specified, multi-dimensional set of criteria encompassing statistical fit, classification reliability, and clinical interpretability.

**Figure 2:**
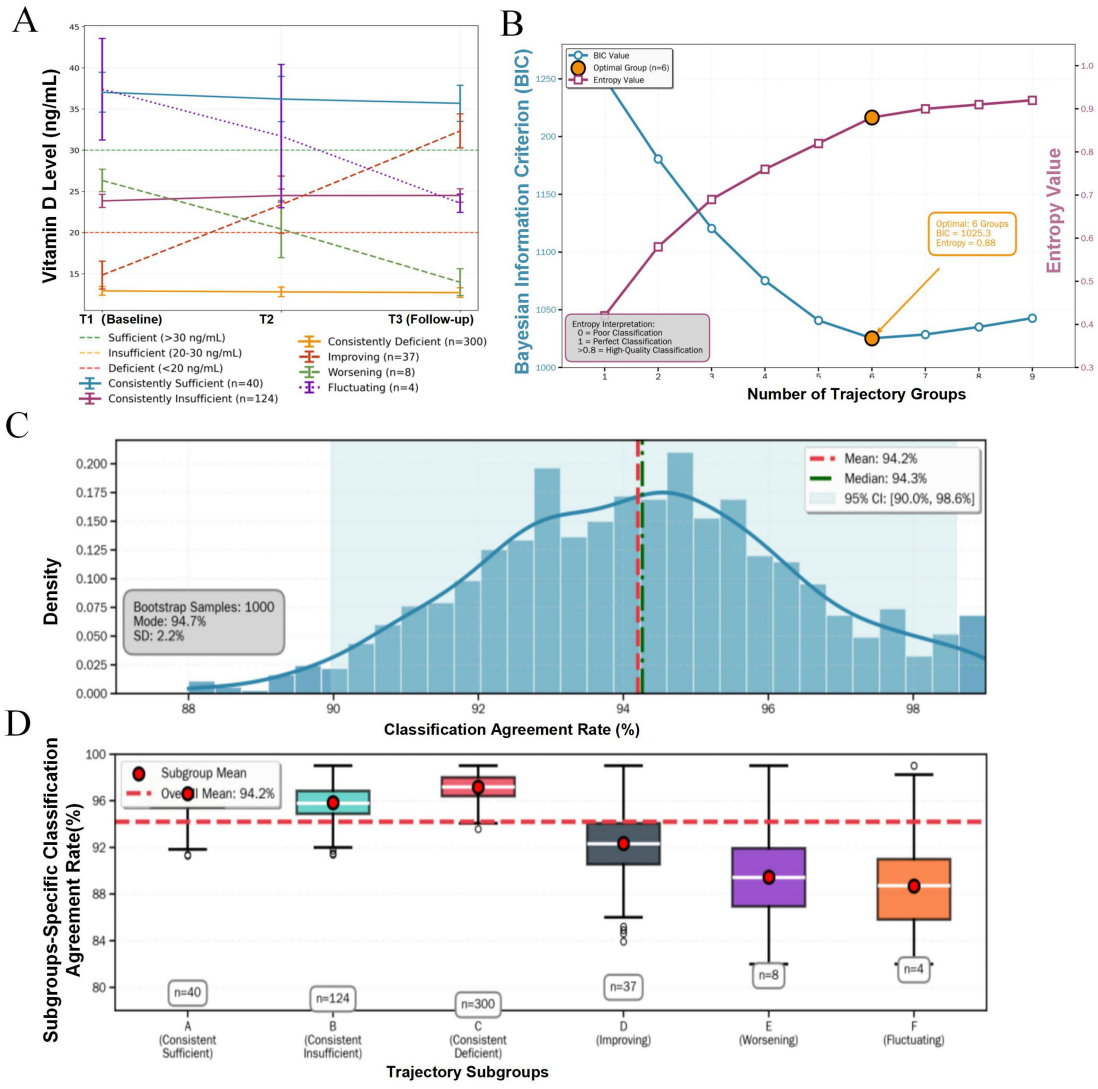
Group-based trajectory model of vitamin D levels. **(A)** Vitamin D levels of 6 trajectory groups identified; **(B)** GBTM model selection criteria for vitamin D trajectory analysis; **(C)** Distribution of overall classification agreement rates (1,000 bootstrap resamples); **(D)** Subgroup-specific classification agreement rates (500 bootstrap resamples per subgroup).

**Figure 3:**
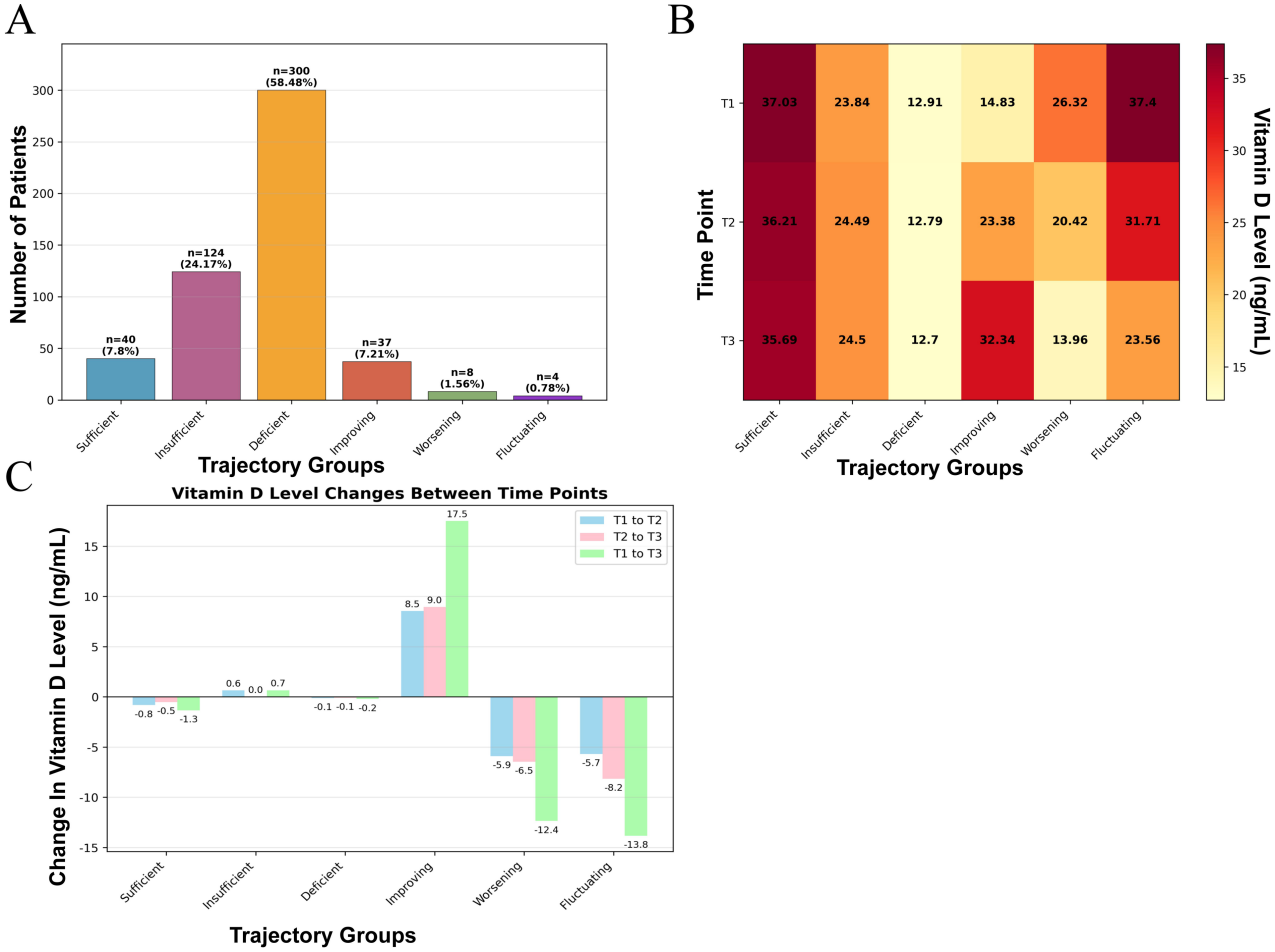
Comprehensive GBTM analysis results for vitamin D levels. **(A)** Distribution of patients across trajectory groups; **(B)** Mean vitamin D levels (ng/mL) by group and time; **(C)** Vitamin D level changes between time points.

Figure 2A illustrates the longitudinal trends of vitamin D levels across the six trajectory subgroups. The 6-subgroup model yielded the lowest BIC value (1025.3) across all evaluated models, providing strong evidence of superior data fit. Furthermore, the model achieved an entropy value of 0.88, which exceeded the widely accepted empirical threshold of 0.8, indicating high accuracy and discriminatory power for individual trajectory classification (Figure 2B). All subgroups exhibited high classification reliability, with an overall APPA of 0.91 (Supplementary Table 2), surpassing the recommended threshold of 0.9. APPA values for individual subgroups ranged from 0.85 to 0.92, with a standard deviation of 0.02 (well below the 0.05 threshold), further confirming the model’s excellent classification performance. Robustness validation further verified the stability of our trajectory classification. Specifically, 1,000 iterations of bootstrap resampling yielded an average classification agreement rate of 94.2% between the resampled models and the original 6-subgroup model (Figure 2C). Sensitivity analyses using fewer trajectory classes further supported the robustness of our core findings. We re-fitted GBTMs specifying 2, 3, 4, and 5 trajectory groups, and compared their fit statistics and prognostic associations with the primary 6-subgroup model. Notably, the 5-group model demonstrated excellent fit (BIC = 1040.8, entropy = 0.82) and produced prognostic associations nearly identical to those of the 6-subgroup model. The six identified subgroups were defined as follows: Consistently Sufficient (*n* = 40), Consistently Insufficient (*n* = 124), Consistently Deficient (*n* = 300), Improving (*n* = 37), Worsening (*n* = 8), and Fluctuating (*n* = 4). Key clinical reference thresholds for vitamin D status are also annotated in the figure for contextual comparison. In-depth analysis of the demographic characteristics of each subgroup revealed that the Consistently Deficient subgroup was the largest, comprising 300 participants (58.48% of the total study population). This high prevalence underscores that vitamin D deficiency is not an isolated finding, but a highly prevalent and clinically relevant health concern within the study cohort (Figure 3).

### 3.3 Association of vitamin D trajectory groups with event-free survival

**Figure 4:**
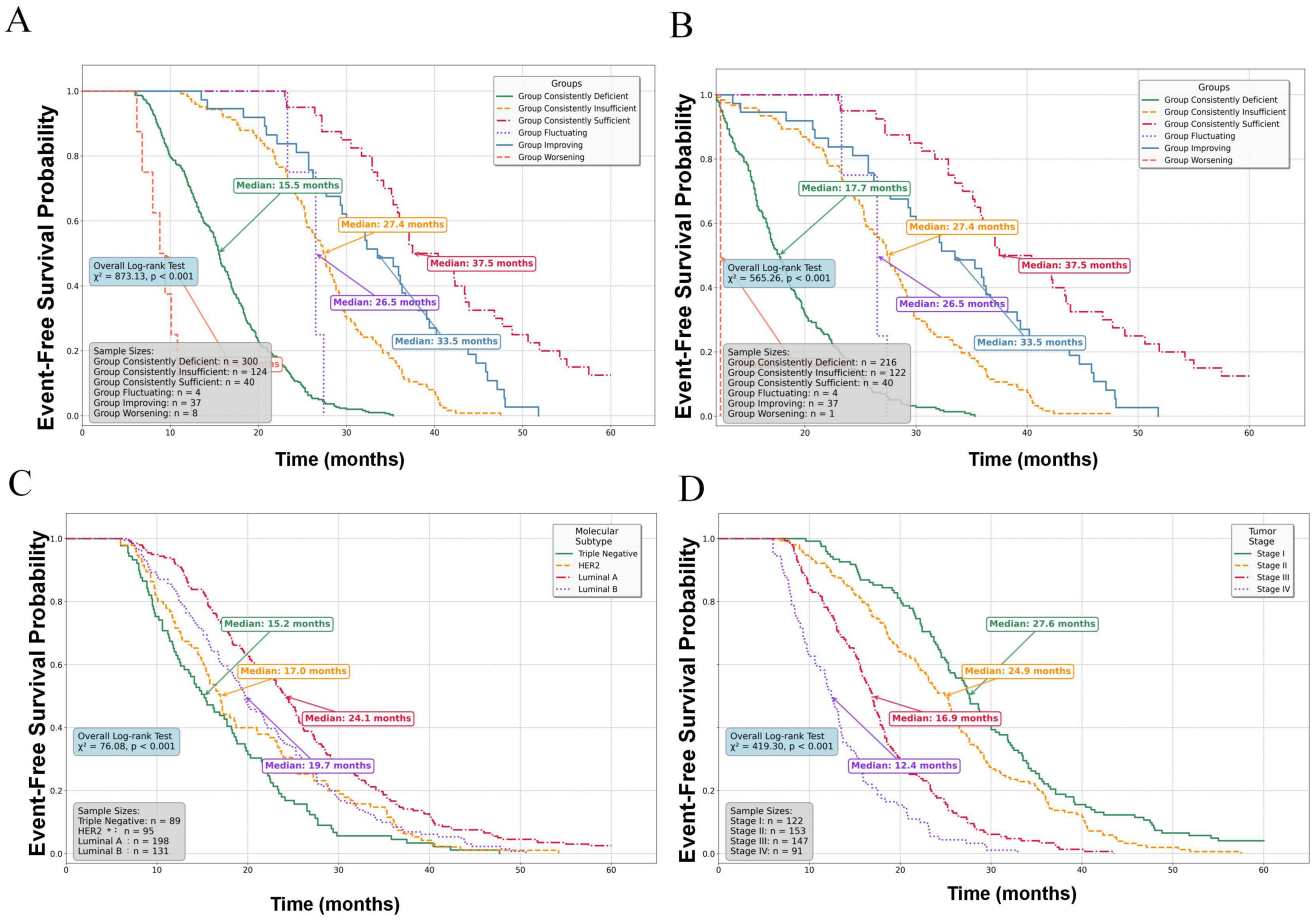
Kaplan–Meier curves of EFS by vitamin D trajectory groups. **(A)** Kaplan–Meier survival curves for EFS (complete dataset, *n* = 513); **(B)** Kaplan–Meier survival curves for EFS (after excluding ≤12-month events, *n* = 420); **(C)** Association of tumor stage and vitamin D trajectory groups with EFS; **(D)** Association of molecular subtype and vitamin D trajectory groups with EFS.

Following the successful identification of six vitamin D dynamic change trajectory subgroups via GBTM, this study further investigated the association between different vitamin D nutritional trajectory groups and event-free survival in breast cancer patients, aiming to clarify whether the dynamic variation patterns of vitamin D serve as an influencing factor for patient prognosis and lay a core foundation for subsequent prognostic analyses. We employed vitamin D trajectory groups as the primary stratification factor, with event-free survival (months) as the endpoint, to plot Kaplan–Meier survival curves and conduct in-depth analysis (Figure 4A). Inter-group comparisons were performed using the Log-rank test, with the Consistently Sufficient group serving as the reference group. Results indicated that, compared with the reference group, patients in the Consistently Deficient group exhibited significantly shorter event-free survival (*p* < 0.001). Patients in the Consistently Insufficient group likewise demonstrated significantly shorter event-free survival compared with the reference group (*p* < 0.001). The difference between the Improving group and the reference group was also significant (*p* = 0.0437), though the effect size was notably lower than that of the Consistently Deficient and Consistently Insufficient groups. Additionally, landmark analysis excluding patients who experienced early EFS events within 12 months of follow-up (*n* = 93) demonstrated that the association between vitamin D trajectories and EFS remained statistically significant, with no material alteration to our core conclusions (Figure 4B). Overall, vitamin D status was significantly associated with patient prognosis: patients in the Consistently Sufficient group had significantly better event-free survival than all other groups. Among them, patients in the Consistently Deficient group had the highest risk of disease progression and the worst clinical prognosis. While the prognosis for patients in the Improving group was inferior to that of the Consistently Sufficient group, it remained superior to that of the Consistently Insufficient group. These results demonstrate that dynamic assessment of vitamin D status holds significant clinical value for predicting patient outcomes.

As tumor stage and molecular subtype are core prognostic determinants for breast cancer, we sequentially stratified the cohort by these two factors to exclude confounding effects, verify the independence of vitamin D trajectory grouping’s prognostic impact. Kaplan–Meier analysis (Figure 4C) revealed a progressive EFS decline from stage I to IV: only the stage I vs. II survival difference was non-significant (*p* = 0.051), with all other stage pairs showing highly significant differences (*p* < 0.001). Across the full cohort and all stage subgroups, Log-rank tests confirmed significant inter-group EFS differences based on vitamin D trajectories (all *p* < 0.001, Supplementary Figure 1): Consistently Sufficient patients had the longest EFS, Consistently Deficient patients had the shortest, and the Improving group (with recovered vitamin D from prior insufficiency/deficiency) outperformed those with persistently abnormal levels. Further stratification by molecular subtype (Luminal A, Luminal B, HER2-positive, TNBC) detected significant survival differences across subtypes (χ^2^ > 100, *p* < 0.001, Figure 4D). Within each subtype, the same trajectory-based EFS pattern was observed (Supplementary Figure 2): the Improving group outperformed Consistently Deficient patients in Luminal A, Luminal B and HER2-positive subtypes. Notably, Luminal A presented the most prominent trajectory-related survival differences, indicating the greatest intervention benefits for this subtype; even for poor-prognosis TNBC, sufficient vitamin D prolonged EFS, supporting its value as an adjuvant strategy. These findings indicate that dynamic monitoring of vitamin D status facilitates prognostic prediction in breast cancer patients, representing a promising strategy for improving long-term outcomes regardless of tumor stage or molecular subtype.

### 3.4 Risk-stratified vitamin D trajectory model for prognostic prediction in breast cancer

Building upon the aforementioned analysis of the association between vitamin D trajectory groups and prognosis, this study aimed to construct a more practical prognostic prediction model for breast cancer. Specifically, we performed risk stratification of vitamin D trajectories based on GBTM results, validated their predictive efficacy, and developed a visual prediction tool to provide a reference for clinical individualized prognostic assessment. The risk stratification framework was pre-specified prior to data analysis, following three core principles: (1) anchoring to the established clinical classification of vitamin D status; (2) ensuring significant prognostic separation between strata; (3) balancing statistical rigor and clinical practicality. Based on these principles, we reclassified the vitamin D trajectories into three categories: low-risk (combining the Consistently Sufficient and Improving trajectories), medium-risk (Consistently Insufficient), and high-risk (combining the Consistently Deficient and Worsening trajectories). The Fluctuating group (*n* = 4) was excluded due to insufficient statistical power.

**Figure 5:**
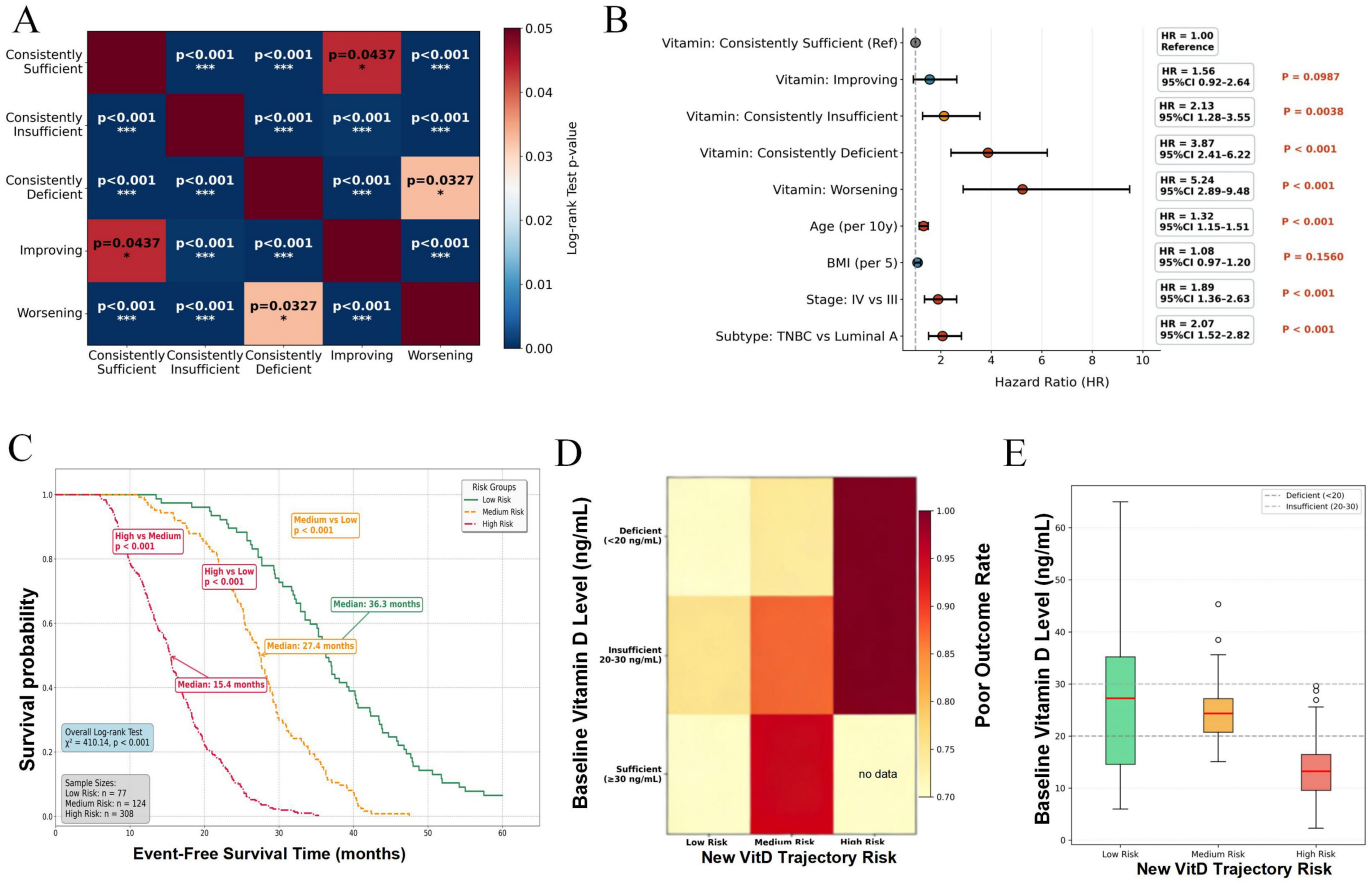
Construction and validation of the vitamin D trajectory-based risk stratification model for EFS in breast cancer patients. **(A)** Pairwise log-rank test heatmap for EFS among vitamin D trajectory subgroups; **(B)** Forest plot of multivariable Cox proportional hazards regression analysis, showing adjusted hazard ratios (HRs) and 95% confidence intervals (CIs) for each original trajectory subgroup, with the Consistently Sufficient group as the reference; **(C)** Kaplan–Meier curves of EFS for the final three-category risk stratification (all pairwise *p* < 0.001); **(D)** Interaction heatmap illustrating the interplay between baseline vitamin D status and the new trajectory-based risk stratification, with the color gradient representing the rate of adverse prognosis events; **(E)** Box plots showing the distribution of baseline vitamin D levels across the three risk strata.

Within the low-risk group, the pairwise log-rank test between the Consistently Sufficient and Improving groups showed a borderline significant difference in EFS (*p* = 0.0437,Figure 5A). However, in the multivariate Cox model adjusted for age, tumor stage, and molecular subtype, the hazard ratio (HR) for the Improving group (vs. Consistently Sufficient group) was 1.56 (95%CI 0.92–2.64, *p* = 0.0987), confirming no statistically significant difference in prognostic risk between the two subgroups(Figure 5B).

For the medium-risk group, the Consistently Insufficient group had significantly worse prognosis than the low-risk group (*p* < 0.001) and significantly better prognosis than the high-risk group (*p* < 0.001). This distinct prognostic separation from both the low-risk and high-risk groups supports its independent classification as the medium-risk category (Figure 5C). Following the exclusion of patients (*n* = 93) who experienced early EFS events within 12 months of follow-up, a landmark analysis was conducted. Results indicated that the association between Vitamin D trajectory risk groups and EFS remained statistically significant (Supplementary Figure 3).

Within the high-risk group, the pairwise log-rank test between the Consistently Deficient and Worsening subgroups showed a weak significant difference in EFS (*p* = 0.0327, Figure 5A). However, in the adjusted multivariate Cox model, both subgroups conferred similarly elevated risks of adverse outcomes: compared with the Consistently Sufficient group, the HR was 3.87 (95%CI: 2.41–6.22) for the Consistently Deficient subgroup and 5.24 (95%CI: 2.89–9.48) for the Worsening subgroup (Figure 5B). Their 95% confidence intervals overlapped substantially, confirming their comparable high-risk characteristics and supporting their combination into a single high-risk category.

This study employed stratified 10-fold cross-validation and a sample splitting method (training set: validation set = 7:3) for internal validation of the prediction model. The results demonstrated consistent discriminatory performance across validation sets, with no evidence of significant overfitting. Detailed results are presented in the Supplementary Table 3. The baseline and new trajectory interaction heatmap illustrates the interplay between baseline vitamin D levels and new trajectory risk (Figure 5D). Patients with high-risk trajectories combined with baseline deficiency had a high adverse prognosis rate; even with sufficient baseline levels, patients with worsening trajectories (high-risk group) still faced a relatively high risk of adverse prognosis. Box plots illustrate the distribution of baseline vitamin D levels across the different risk groups (Figure 5E). The low-risk group had significantly higher baseline vitamin D levels than the medium/high-risk groups, and baseline vitamin D in the high-risk group was mostly concentrated in the deficient range (<20 ng/mL).

### 3.5 Development and validation of a prognostic nomogram integrating vitamin D trajectory risk group for EFS

**Figure 6:**
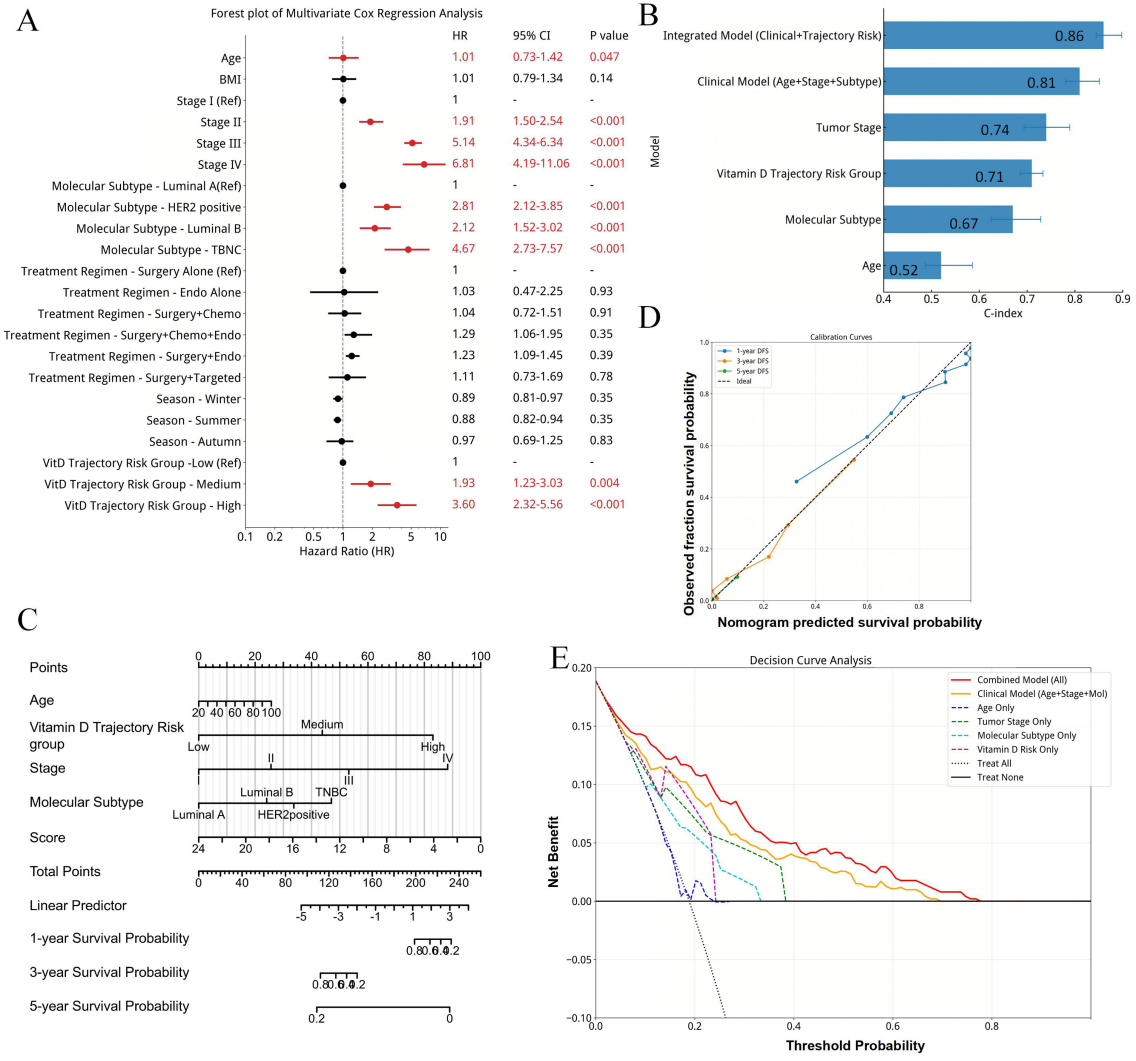
Prognostic performance evaluation and individualized risk prediction model development based on the integrated model incorporating vitamin D trajectory risk group. **(A)** Forest plot of multivariate Cox proportional hazards regression analysis identifying independent prognostic factors for EFS; **(B)** C-index across distinct prognostic models to assess discriminative ability; **(C)** The comprehensive prognostic nomogram constructed with the final independent predictors (age, tumor stage, molecular subtype, and vitamin D trajectory risk group), enabling individualized estimation of EFS probabilities; **(D)** Calibration curves of the nomogram. The dashed line represents an ideal perfect prediction, while the solid line depicts the actual performance of the nomogram, revealing high consistency between the nomogram-predicted survival probabilities and the observed clinical outcomes; **(E)** DCA of the nomogram. The y-axis indicates the clinical net benefit, and the x-axis represents the threshold probability.

The proportional hazards assumption of the Cox model was verified using Schoenfeld residuals, with all variables meeting the assumption (all *p* > 0.05, Supplementary Figure 4). Covariates were selected based on clinical relevance and univariate analysis (*p* < 0.1), followed by backward stepwise selection; final multivariate models included age, tumor stage, molecular subtype, and vitamin D trajectory risk group. After adjustment, vitamin D trajectory risk group remained an independent prognostic factor for EFS (high-risk vs. low-risk: HR = 3.60, 95% CI: 2.32–5.56, *p* < 0.001) (Figure 6A). The Cox proportional hazards regression model was used to evaluate model performance. Results indicated that the vitamin D risk-trajectory-based single-factor model achieved a C-index of 0.71, exceeding the 0.7 threshold, this suggests robust discriminative ability. Notably, while the clinical model incorporating factors such as age, tumor stage, and molecular subtype yielded a baseline C-index of 0.81, the addition of vitamin D trajectory risk group further elevated this value to 0.86, demonstrating enhanced predictive performance (Figure 6B).

Subsequently, we incorporated key predictive factors including age, tumor stage, molecular subtypes, and the new vitamin D risk trajectory group (low to high) to develop a comprehensive risk prediction nomogram to facilitate efficient estimation of individual prognostic risk (Figure 6C). The calibration curve demonstrates a high degree of agreement between the survival probability predicted by the nomogram and the observed outcomes, indicating that the model has good calibration (Figure 6D).

Decision curve analysis (DCA) demonstrated that, our integrated prognostic model, incorporating vitamin D trajectory risk group, age, tumor stage, and molecular subtype, demonstrated broad clinical applicability with positive net clinical benefit across a threshold probability range of approximately 10 to 65%, which encompasses nearly all clinically relevant thresholds for risk-based interventions in breast cancer management. More importantly, compared with the standard clinical model comprising only traditional prognostic factors (age, tumor stage, and molecular subtype), our integrated model consistently achieved superior net clinical benefit across the entire range of threshold probabilities. These findings collectively indicate that integrating dynamic vitamin D trajectory information into prognostic models provides incremental value over standard clinical models, enabling more accurate patient risk stratification, enhanced clinical decision-making, and ultimately improved personalized breast cancer care (Figure 6E). Of note, these findings regarding the calibration and clinical net benefit of the model are currently based on our single-center cohort. Before this model can be widely applied in routine clinical practice, further external validation in independent multi-center cohorts is warranted to confirm its generalizability and clinical applicability.

## 4 Discussion

This retrospective cohort study is the first to systematically characterize six distinct dynamic trajectories of serum vitamin D levels in 513 breast cancer patients using GBTM and to comprehensively evaluate their prognostic impact on EFS. Our key findings confirm that dynamic vitamin D trajectories are a robust, independent prognostic factor for breast cancer, outperforming static single measurements in prognostic value. Furthermore, we developed a visual nomogram to facilitate clinical translation. These results highlight the clinical significance of longitudinal vitamin D monitoring in breast cancer patients and provide a novel nutritional perspective for personalized prognostic assessment and intervention.

A striking finding of our study is the high prevalence of vitamin D deficiency in the breast cancer cohort: 64.9% of patients presented with severe vitamin D deficiency at baseline, and 89.5% had either deficiency or insufficiency, requiring nutritional supplementation. This is consistent with previous reports of high vitamin D deficiency rates in cancer populations (21), which may be attributed to multiple factors including reduced sun exposure (a primary source of endogenous vitamin D synthesis), malnutrition related to cancer cachexia or treatment-related adverse effects, impaired vitamin D metabolism in cancer patients, and potential sequestration of vitamin D by tumor tissues (22–24). Notably, the Consistently Deficient group accounted for 58.48% of the total population—the largest of the six trajectory subgroups—indicating that persistent vitamin D deficiency is a common clinical issue in breast cancer patients and underscoring the need for routine vitamin D screening and targeted supplementation in this population.

Our survival analyses demonstrated a clear and graded association between vitamin D trajectories and EFS: the Consistently Sufficient group had the most favorable prognosis, followed by the Improving group (intermediate risk), while the Consistently Deficient and Consistently Insufficient groups had significantly worse EFS (*p* < 0.001) compared to the Consistently Sufficient group. This finding aligns with the anti-tumor mechanisms of vitamin D and suggests that sustained adequate vitamin D status is critical for favorable breast cancer outcomes, while even partial improvement in vitamin D status (the Improving group) confers prognostic benefits relative to persistent insufficiency or deficiency. The intermediate prognostic risk of the Improving group further supports the potential value of vitamin D supplementation interventions: correcting vitamin D deficiency/insufficiency during treatment may reverse the adverse prognostic impact of suboptimal vitamin D status, a finding with direct clinical implications for nutritional intervention strategies.

Importantly, the prognostic association between vitamin D trajectories and EFS remained significant after stratification by tumor stage and molecular subtype—two established core prognostic factors for breast cancer (25–28). In all tumor stage subgroups (I-IV) and all molecular subtype subgroups (Luminal A, Luminal B, HER2-positive, triple-negative breast cancer [TNBC]), the Consistently Sufficient group exhibited the longest EFS, and the Consistently Deficient group had the shortest. This confirms the independence of vitamin D trajectories as a prognostic factor, indicating that its impact on breast cancer progression is not confounded by tumor burden or biological subtype. For Luminal A patients—the subtype with the best overall prognosis (29)—we observed the most prominent survival advantage in the Consistently Sufficient group, suggesting that vitamin D monitoring and intervention may yield the greatest clinical benefits in this population. Even for TNBC patients—the subtype with the worst prognosis (30)—sustained vitamin D sufficiency was associated with prolonged EFS, highlighting the potential of vitamin D as an adjuvant prognostic and therapeutic target for this clinically challenging subtype.

To translate these findings into clinical practice, we reclassified the six vitamin D trajectories into three clinically actionable risk groups, namely low, medium, and high, establishing a new risk prediction model. The C-index of this model was 0.71, indicating good predictive accuracy and representing a significant improvement compared to models based solely on static vitamin D measurements. The visual nomogram we developed allows for rapid, individualized prognostic risk calculation by assigning weighted scores to each predictor, making it a practical tool for clinicians to conduct bedside risk assessment and guide treatment decisions. Our analysis of the interaction between baseline vitamin D and trajectory risk further revealed that even patients with sufficient baseline vitamin D face an increased risk of adverse outcomes if their vitamin D status worsens over time (Worsening group), emphasizing that longitudinal monitoring—rather than a single baseline measurement—is essential for accurate prognostic assessment.

### 4.1 Limitations and future research directions

This study provides novel insights into the prognostic value of dynamic vitamin D trajectories in breast cancer, but several limitations must be acknowledged, with corresponding future research directions proposed to address these gaps.

#### 4.1.1 Study design and biases

This single-center retrospective cohort study is inherently limited by information bias from electronic medical record documentation inconsistencies, and selection/survival bias from strict inclusion criteria requiring complete serial vitamin D measurements, which may overrepresent patients with favorable clinical characteristics and underestimate the adverse prognostic impact of persistent vitamin D deficiency. Additionally, the cohort was limited to Northeast Chinese patients, restricting generalizability to other ethnic groups and healthcare settings.

Future Directions: Conduct large-scale multicenter prospective cohort studies with standardized data collection protocols, and apply inverse probability of treatment weighting to adjust for selection bias.

#### 4.1.2 Follow-up duration and censoring

Higher-frequency serial testing enables a more comprehensive depiction of vitamin D dynamic alterations. Nonetheless, the median follow-up of 38.0 months is insufficient to capture late recurrence events (10–15 years) in estrogen receptor-positive Luminal subtypes, limiting the assessment of long-term prognostic effects. The 72.5% overall censoring rate may introduce non-random censoring bias, potentially underestimating the hazard ratio for adverse outcomes in high-risk trajectory groups.

Future Directions: Extend follow-up to a minimum of 10 years, establish a centralized long-term follow-up database, and use weighted Cox proportional hazards models to account for non-random censoring.

#### 4.1.3 Confounding factors

Detailed data on post-enrollment vitamin D supplementation (dosage, duration, adherence) were not collected, precluding disentanglement of the independent prognostic effect of trajectories from supplementation itself. Interactions between vitamin D trajectories and systemic therapies (endocrine, targeted, immunotherapy) were not explored, and lifestyle confounders (diet, sun exposure) were not controlled.

Future Directions: Prospectively collect standardized supplementation and lifestyle data, and perform stratified analyses to evaluate treatment-trajectory interactions.

#### 4.1.4 Methodological limitations

Some trajectory subgroups (e.g., Worsening group, *n* < 10) had small sample sizes, reducing statistical power for subgroup analyses. The prognostic nomogram only underwent internal validation, and GBTM subgroup selection has inherent subjectivity.

Future Directions: Our preliminary, hypothesis-generating findings require further validation through expanded cohort studies to capture rare trajectory subgroups, external validation in independent populations, and head-to-head comparison of GBTM and latent class growth mixed modeling (LCGMM) to confirm trajectory robustness. Large-scale prospective studies and randomized controlled trials are essential before longitudinal vitamin D monitoring can be integrated into routine clinical practice for breast cancer patients.

#### 4.1.5 Outcome measure limitations

This study exclusively used EFS as the primary endpoint, failing to distinguish between local recurrence and distant metastasis, and did not evaluate overall survival, distant metastasis-free survival, treatment-related toxicities, or patient quality of life.

Future Directions: Incorporate multiple clinically relevant endpoints, apply competing risk models for event-specific analysis, and collect data on treatment adverse events and patient-reported outcomes.

#### 4.1.6 Causal inference limitations

As an observational study, causal relationships cannot be established. Residual confounding from unmeasured factors (genetic polymorphisms, tumor genomic features) and potential reverse causality (aggressive tumors disrupting vitamin D metabolism) remain. The underlying molecular mechanisms linking trajectories to prognosis were not elucidated.

Future Directions: Conduct randomized controlled trials to test personalized vitamin D supplementation strategies, perform Mendelian randomization to minimize confounding, integrate multi-omics approaches to explore molecular mechanisms. Future studies can further integrate imaging biomarkers into our current prognostic model, which may further improve the predictive performance of the model, and provide more comprehensive personalized assessment for breast cancer patients (31).

### 4.2 Clinical implications

Our findings generate important hypotheses that could hold future clinical implications for the management of breast cancer patients, though further validation is required before these strategies can be routinely implemented into standard clinical care. First, given the high prevalence of vitamin D deficiency we observed in this population, our results highlight that screening for serum vitamin D levels could be a valuable target for future clinical practice. Second, our data suggest that longitudinal vitamin D monitoring (at baseline, mid-treatment, and post-treatment) may provide additional prognostic value beyond single measurements, which appear insufficient to fully characterize an individual’s dynamic risk trajectory. While these findings are hypothesis-generating, trajectory-based vitamin D assessment remains investigational at this stage, and future studies are needed to confirm whether such monitoring can be effectively translated into routine clinical practice. Third, for patients with deficient or insufficient vitamin D status, targeted supplementation with regular monitoring to assess effectiveness and adjust dosages to achieve and maintain sufficiency could be considered as a potential nutritional strategy. Fourth, the risk stratification model and nomogram developed in this study may serve as an exploratory tool for individualized prognostic assessment. These tools could help to identify high-risk patients who might benefit from more intensive surveillance and intervention in future research settings, laying a foundation for guiding personalized treatment plans once their clinical performance is externally validated.

## 5 Conclusion

In summary, this study demonstrates that dynamic serum vitamin D trajectories are a strong, independent prognostic factor for breast cancer, with sustained vitamin D sufficiency associated with favorable EFS and persistent deficiency linked to poor outcomes. The vitamin D trajectory risk stratification model exhibits good predictive performance, and the visual nomogram provides a practical tool for clinical prognostic assessment. Our findings identify a potential relationship between serial vitamin D measurements and breast cancer clinical parameters. These observations do not yet support routine clinical implementation but provide a rationale for future prospective studies to determine whether vitamin D status assessment could contribute to personalized risk stratification and patient management. Future RCTs are needed to confirm the causal relationship between vitamin D supplementation and improved breast cancer outcomes, and to establish evidence-based guidelines for vitamin D screening and intervention in this population.

## Data Availability

The original contributions presented in the study are included in the article/Supplementary material, further inquiries can be directed to the corresponding author/s.

## Supplementary material

SUPPLEMENTARY FIGURE 1 Kaplan-Meier curves of event-free survival by vitamin D trajectory group: Stage I **(A)**; Stage II **(B)**; Stage III **(C)**; Stage IV **(D).**

SUPPLEMENTARY FIGURE 2. Kaplan-Meier curves of event-free survival by vitamin D trajectory group: Luminal A **(A)**; Luminal B **(B)**; HER-2 positive **(C)**; TNBC **(D)**.

SUPPLEMENTARY FIGURE 3. Kaplan-Meier curves for EFS stratified by vitamin D trajectory risk groups. A total of 420 patients were included after excluding 93 patients with EFS events within the first 12 months of follow-up. Log-rank test was used to compare EFS between groups.

SUPPLEMENTARY FIGURE 4 Schoenfeld residual plots for proportional hazards assumption, all variables meeting the assumption (all *p*>0.05)
