# Supplementary figures and images for: Dynamic vitamin D trajectories and their prognostic value in breast cancer: a group-based trajectory modeling study
Source: Front Nutr. 2026 Jun 4;13:1839196. doi: 10.3389/fnut.2026.1839196 (PMC13275254; doi:10.3389/fnut.2026.1839196)

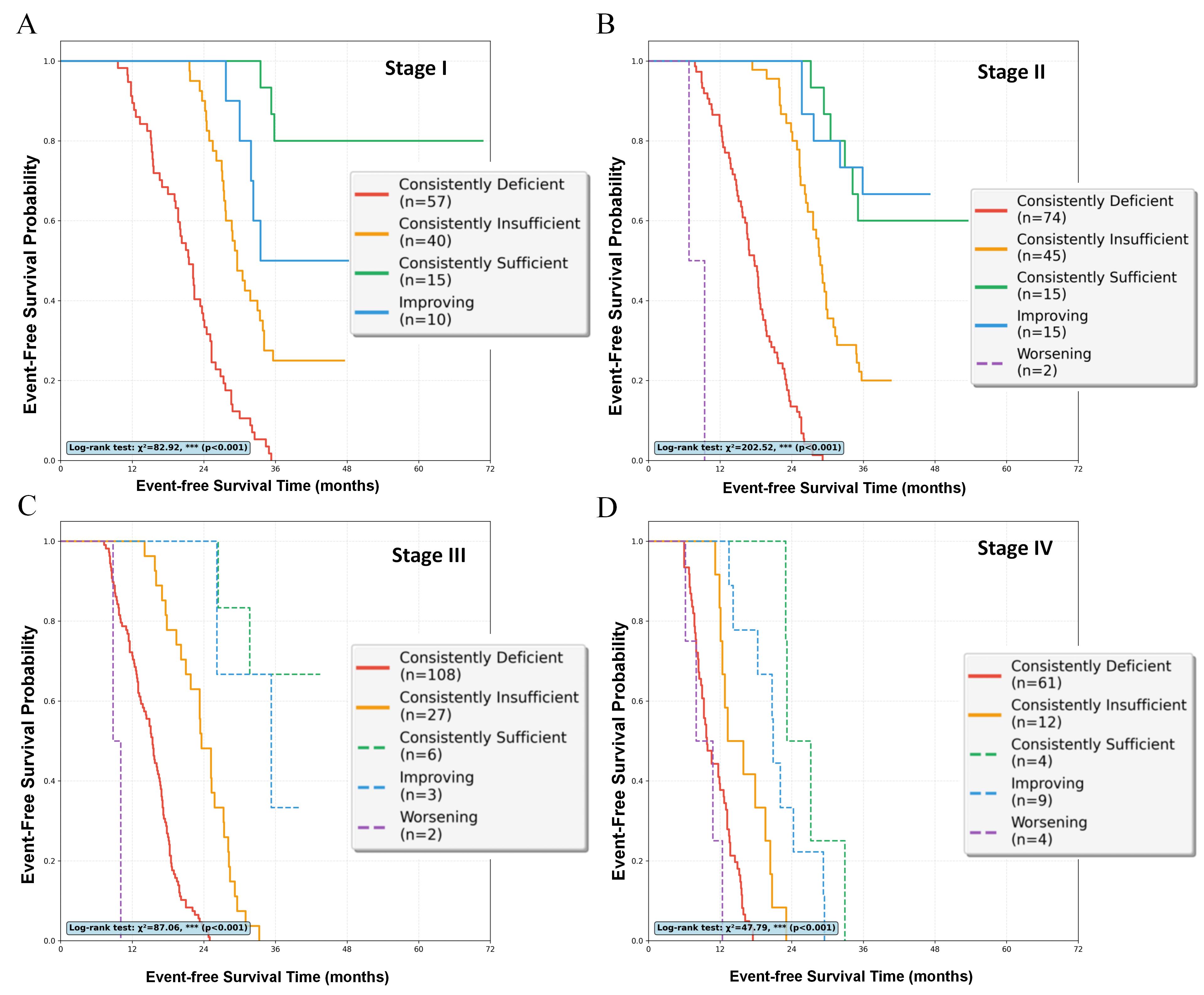

Supplement: SUPPLEMENTARY FIGURE 1 — Kaplan-Meier curves of event-free survival by vitamin D trajectory group: Stage I (A); Stage II (B); Stage III (C); Stage IV (D). [file Image_1.JPEG]

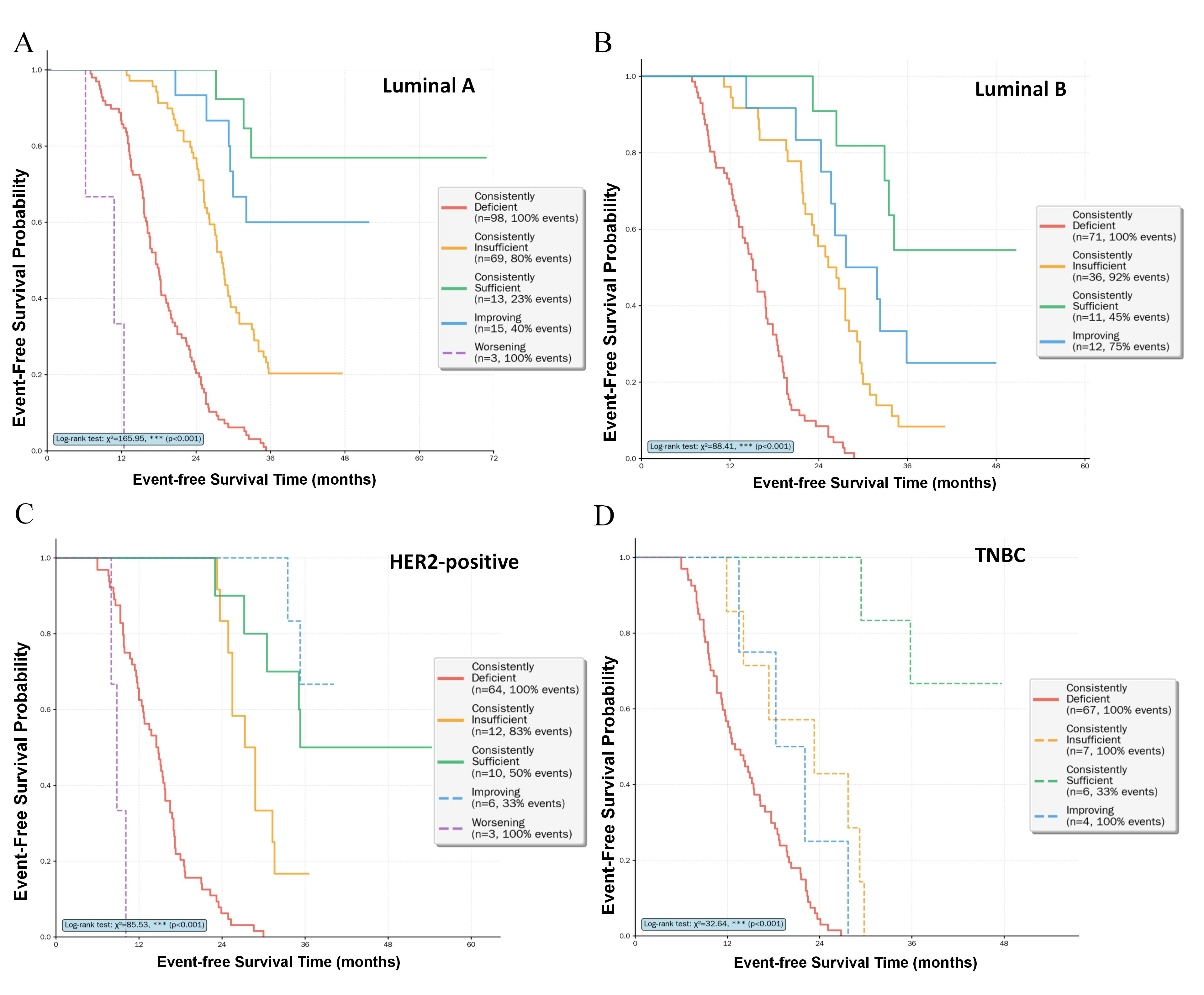

Supplement: SUPPLEMENTARY FIGURE 2. — Kaplan-Meier curves of event-free survival by vitamin D trajectory group: Luminal A (A); Luminal B (B); HER-2 positive (C); TNBC (D). [file Image_2.JPEG]

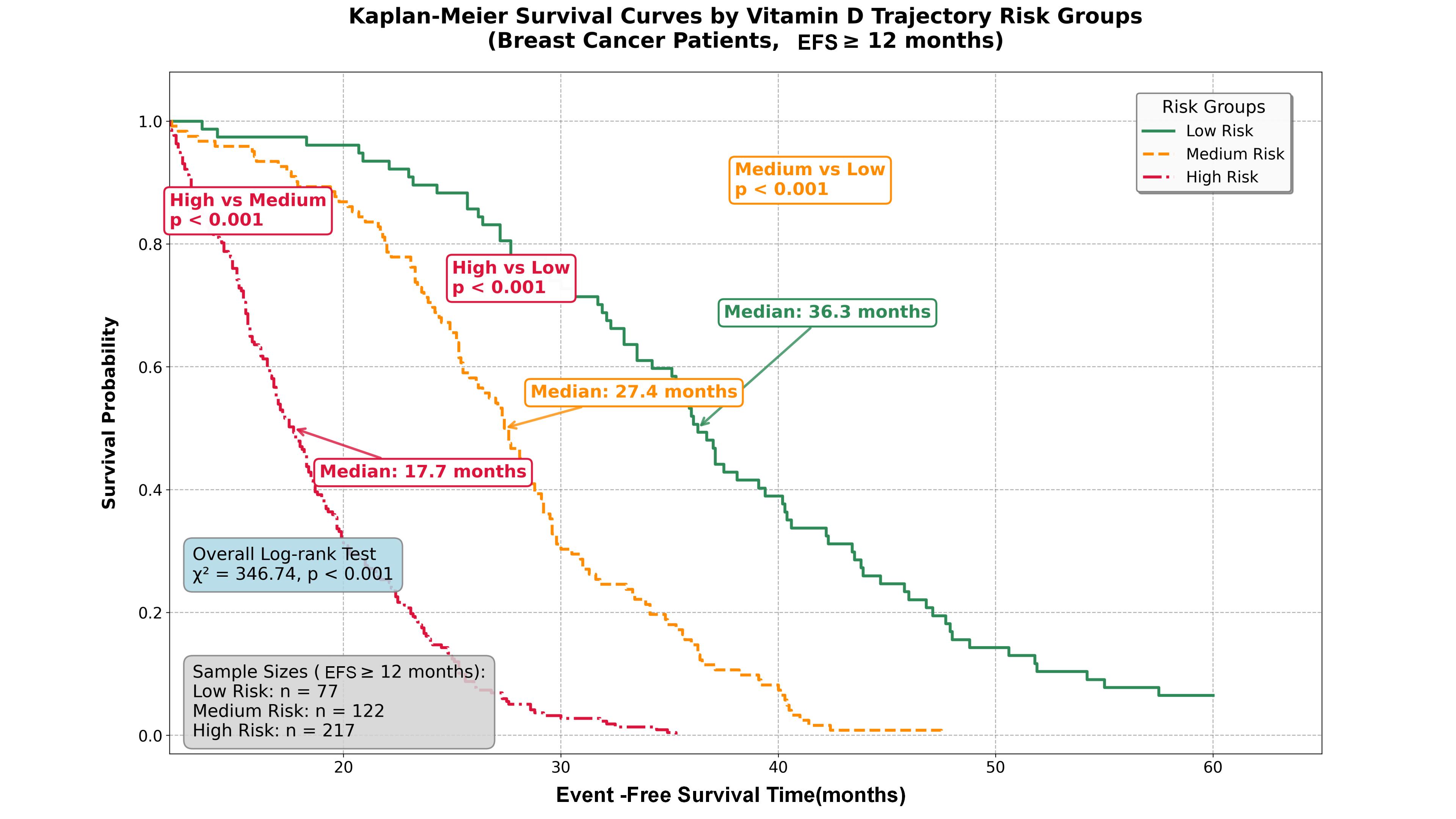

Supplement: SUPPLEMENTARY FIGURE 3. — Kaplan-Meier curves for EFS stratified by vitamin D trajectory risk groups. A total of 420 patients were included after excluding 93 patients with EFS events within the first 12 months of follow-up. Log-rank test was used to compare EFS between groups. [file Image_3.JPEG]

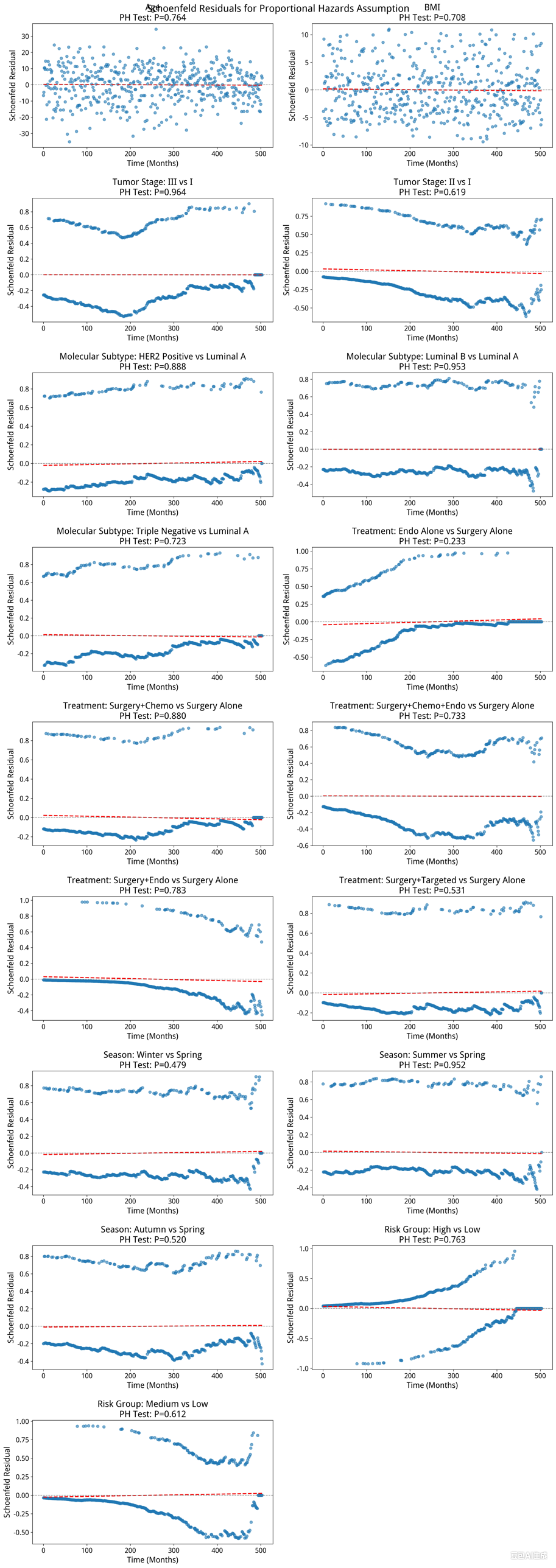

Supplement: SUPPLEMENTARY FIGURE 4 — Schoenfeld residual plots for proportional hazards assumption, all variables meeting the assumption (all p>0.05) [file Image_4.JPEG]
