# Supplementary material for: Dynamic vitamin D trajectories and their prognostic value in breast cancer: a group-based trajectory modeling study
Source: Front Nutr. 2026 Jun 4;13:1839196. doi: 10.3389/fnut.2026.1839196 (PMC13275254; doi:10.3389/fnut.2026.1839196)
Supplement: Supplementary file 5 [file Table_1.DOCX]

**Supplementary Table 1**

**Table 1.1 Baseline characteristics of the study cohort and patients excluded due to incomplete vitamin D data.**

| **Variable** | **Clinic Cohort**  **(n = 513)** | **Excluded Patients (n = 127)** | ***P*-value** |
| --- | --- | --- | --- |
| Gender,n(%) |  |  |  |
| Female | 513(100%) | 127 (100%) | N/A |
| Age, mean (SD) [range] years | 55.8 ± 10.9 [20-91] | 56.5 ± 11.2 [22–88] | 0.526 |
| Body mass index, mean (SD) [range] kg/m2 | 25.0 ± 4.6 [16.1-35.8] | 24.7 ± 4.5 [17.3–36.2] | 0.483 |
| Tumor Stage,n(%) |  |  | 0.512^#^ |
| Stage I | 122 (23.8%) | 38 (29.9%) |  |
| Stage II | 153 (29.8%) | 33 (26.0%) |  |
| Stage III | 147 (28.7%) | 31 (24.4%) |  |
| Stage IV | 91 (17.7%) | 25 (19.7%) |  |
| Molecular Subtype,n(%) |  |  | 0.698^#^ |
| Luminal A | 198 (38.6%) | 42 (33.1%) |  |
| Luminal B | 131 (25.5%) | 34 (26.8%) |  |
| HER2-positive | 95 (18.5%) | 26 (20.5%) |  |
| Triple-negative breast cancer, TNBC | 89 (17.3%) | 25 (19.7%) |  |

*Continuous variables were presented as mean ± standard deviation and compared using independent samples t-test. Categorical variables were presented as number (percentage) and compared using Pearson χ² test^#^ or Fisher’s exact test where appropriate.*

**Table 1.2 Baseline Characteristics Between Clinic Cohort and Lost-to-Follow-Up Cohort**

| **Variable** | **Clinic Cohort**  **(n = 513)** | **Lost-to-Follow-Up (n=216)** | ***P*-value** |
| --- | --- | --- | --- |
| Gender,n(%) |  |  |  |
| Female | 513(100%) | 216 (100.0%) | N/A |
| Age, mean (SD) [range] years | 55.8 ± 10.9 [20-91] | 56.3 ± 11.1 [21–89] | 0.672 |
| Body mass index, mean (SD) [range] kg/m2 | 25.0 ± 4.6  [16.1-35.8] | 25.2 ± 4.8  [16.2–36.1] | 0.635 |
| Tumor Stage,n(%) |  |  | 0.726^#^ |
| Stage I | 122 (23.8%) | 48 (22.2%) |  |
| Stage II | 153 (29.8%) | 68 (31.5%) |  |
| Stage III | 147 (28.7%) | 59 (27.3%) |  |
| Stage IV | 91 (17.7%) | 41 (19.0%) |  |
| Molecular Subtype,n(%) |  |  | 0.813^#^ |
| Luminal A | 198 (38.6%) | 77 (35.6%) |  |
| Luminal B | 131 (25.5%) | 59 (27.3%) |  |
| HER2-positive | 95 (18.5%) | 42 (19.4%) |  |
| Triple-negative breast cancer, TNBC | 89 (17.3%) | 38 (17.6%) |  |

*Continuous variables were presented as mean ± standard deviation and compared using independent samples t-test. Categorical variables were presented as number (percentage) and compared using Pearson χ² test^#^ or Fisher’s exact test where appropriate.*
