# Supplementary material for: Dynamic vitamin D trajectories and their prognostic value in breast cancer: a group-based trajectory modeling study
Source: Front Nutr. 2026 Jun 4;13:1839196. doi: 10.3389/fnut.2026.1839196 (PMC13275254; doi:10.3389/fnut.2026.1839196)
Supplement: Supplementary file 6 [file Table_2.DOCX]

**Supplementary table 2**

**GBTM Model Selection Criteria**

| Number of Trajectory Groups | BIC Value | Log BF  (2ΔBIC^#^) | Entropy Value | APPA Mean | APPA Std | APPA Range |
| --- | --- | --- | --- | --- | --- | --- |
| 2 | 1180.7 | 310.8 | 0.58 | 0.56 | 0.06 | 0.51-0.65 |
| 3 | 1120.5 | 190.4 | 0.69 | 0.71 | 0.05 | 0.63-0.75 |
| 4 | 1075.2 | 99.8 | 0.76 | 0.78 | 0.04 | 0.72-0.80 |
| 5 | 1040.8 | 31 | 0.82 | 0.84 | 0.03 | 0.79-0.85 |
| 6 | 1025.3 | 0 | 0.88 | 0.91 | 0.02 | 0.85-0.92 |
| 7 | 1028.6 | 6.6 | 0.90 | 0.92 | 0.02 | 0.86-0.93 |

#: 0 = Optimal model (reference);>6 = Positive evidence of inferiority;>10 = Extreme evidence of inferiority.
