# Supplementary material for: Dynamic vitamin D trajectories and their prognostic value in breast cancer: a group-based trajectory modeling study
Source: Front Nutr. 2026 Jun 4;13:1839196. doi: 10.3389/fnut.2026.1839196 (PMC13275254; doi:10.3389/fnut.2026.1839196)
Supplement: Supplementary file 7 [file Table_3.DOCX]

**Supplement Table 3, Internal Validation of the New Risk Model: Stratified 10-Fold Cross-Validation and 7:3 Holdout Validation**

**Table 3.1, The stratified 10-fold cross-validation yielded stable and reliable performance across all iterations**

| Metric | Mean ± Standard Deviation (10 folds) | Performance of the Full Model (Reference) |
| --- | --- | --- |
| C-index | 0.708 ± 0.032 | 0.712 |
| Log-rank Test p-value | <0.001 (all 10 folds) | <0.001 |
| Low-risk Recurrence Rate (%) | 6.3 ± 1.8 | 6.5 |
| Medium-risk Recurrence Rate (%) | 1.7 ± 0.9 | 1.6 |
| High-risk Recurrence Rate (%) | 28.9 ± 2.1 | 29.5 |

**Table 3.2, The holdout validation demonstrated strong generalizability of the new risk model**

| Metric | Training Set (n=359) | Internal Validation Set (n=154) | Absolute Difference |
| --- | --- | --- | --- |
| C-index | 0.715 | 0.702 | 0.013 |
| Log-rank Test p-value | <0.001 | <0.001 | N/A |
| Low-risk Recurrence Rate (%) | 6.7 | 5.9 | 0.80% |
| Medium-risk Recurrence Rate (%) | 1.6 | 1.8 | 0.20% |
| High-risk Recurrence Rate (%) | 29.8 | 28.7 | 1.10% |
